# Molecular interactions of adaptor protein PSTPIP2 control neutrophil-mediated responses leading to autoinflammation

**DOI:** 10.3389/fimmu.2022.1035226

**Published:** 2022-12-20

**Authors:** Nataliia Pavliuchenko, Iris Duric, Jarmila Kralova, Matej Fabisik, Frantisek Spoutil, Jan Prochazka, Petr Kasparek, Jana Pokorna, Tereza Skopcova, Radislav Sedlacek, Tomas Brdicka

**Affiliations:** ^1^ Laboratory of Leukocyte Signalling, Institute of Molecular Genetics of the Czech Academy of Sciences, Prague, Czechia; ^2^ Department of Cell Biology, Charles University, Faculty of Science, Prague, Czechia; ^3^ Czech Centre for Phenogenomics, Institute of Molecular Genetics of the Czech Academy of Sciences, Vestec, Czechia; ^4^ Laboratory of Transgenic Models of Diseases, Institute of Molecular Genetics of the Czech Academy of Sciences, Vestec, Czechia

**Keywords:** neutrophils, autoinflammation, chronic multifocal osteomyelitis, PSTPIP2, PEST-family phosphatases, SHIP1

## Abstract

**Introduction:**

Autoinflammatory diseases are characterized by dysregulation of innate immune system leading to spontaneous sterile inflammation. One of the well-established animal models of this group of disorders is the mouse strain *Pstpip2^cmo^*. In this strain, the loss of adaptor protein PSTPIP2 leads to the autoinflammatory disease chronic multifocal osteomyelitis. It is manifested by sterile inflammation of the bones and surrounding soft tissues of the hind limbs and tail. The disease development is propelled by elevated production of IL-1β and reactive oxygen species by neutrophil granulocytes. However, the molecular mechanisms linking PSTPIP2 and these pathways have not been established. Candidate proteins potentially involved in these mechanisms include PSTPIP2 binding partners, PEST family phosphatases (PEST-PTPs) and phosphoinositide phosphatase SHIP1.

**Methods:**

To address the role of these proteins in PSTPIP2-mediated control of inflammation, we have generated mouse strains in which PEST-PTP or SHIP1 binding sites in PSTPIP2 have been disrupted. In these mouse strains, we followed disease symptoms and various inflammation markers.

**Results:**

Our data show that mutation of the PEST-PTP binding site causes symptomatic disease, whereas mice lacking the SHIP1 interaction site remain asymptomatic. Importantly, both binding partners of PSTPIP2 contribute equally to the control of IL-1β production, while PEST-PTPs have a dominant role in the regulation of reactive oxygen species. In addition, the interaction of PEST-PTPs with PSTPIP2 regulates the production of the chemokine CXCL2 by neutrophils. Its secretion likely creates a positive feedback loop that drives neutrophil recruitment to the affected tissues.

**Conclusions:**

We demonstrate that PSTPIP2-bound PEST-PTPs and SHIP1 together control the IL-1β pathway. In addition, PEST-PTPs have unique roles in the control of reactive oxygen species and chemokine production, which in the absence of PEST-PTP binding to PSTPIP2 shift the balance towards symptomatic disease.

## 1 Introduction

Chronic recurrent multifocal osteomyelitis (CRMO) is an autoinflammatory disease characterized by the development of sterile inflammatory lesions in the bones. Treatment strategies include various ways to suppress inflammation. However, they often fail to induce long-term remission and in many patients the disease relapses (1–3). In part, this is the consequence of the fact that the molecular mechanisms and genetic causes of this disease are poorly understood. To gain better insight into the mechanisms driving CRMO development, several mouse models have been generated. One of the best studied is chronic multifocal osteomyelitis (CMO) mouse model *Pstpip2^cmo^*, which develops sterile bone inflammation in the hind feet and tail (4). The disease is caused by a point mutation in the *Pstpip2* gene, which results in a complete loss of detectable expression of the adaptor protein PSTPIP2 (proline-serine-threonine phosphatase-interacting protein 2) (5, 6). The development of osteomyelitis in the *Pstpip2^cmo^* mice is hematopoietically driven and occurs in the absence of lymphocytes, consistent with an autoinflammatory mechanism of the disease (6). However, similar to human CRMO, the signaling and inflammatory pathways critical for CMO in mice are incompletely understood. Previous studies identified neutrophil granulocytes as a crucial cell type critical for the disease development in *Pstpip2^cmo^* mice (7, 8). They display pathological hyperactivity of the pathways regulating production of active IL-1β and reactive oxygen species (ROS). While IL-1β triggers autoinflammation, ROS production is critical for the bone damage (7–11). Little is known about the molecular mechanisms connecting PSTPIP2 to these pathways.

PSTPIP2 interacts with several regulators of signaling. The most prominent include PEST-family protein tyrosine phosphatases (PEST-PTPs) and Src homology 2-domain–containing inositol 5-phosphatase 1 (SHIP1), binding of which is dependent on W232 and phosphorylated C-terminal tyrosines of PSTPIP2, respectively (12–14). These proteins are the best candidates, through which PSTPIP2 could negatively regulate pro-inflammatory signaling. The family of PEST-PTPs has three members, PTPN12 (PTP-PEST), PTPN22 (LYP/PEP), and PTPN18 (BDP1/PTP-HSCF), which all interact with PSTPIP2 (14, 15). While little is known about the roles of PTPN12 and PTPN18 in neutrophils, deficiency in PTPN22 was shown to impair neutrophil functions triggered by Fc receptor stimulation, including adhesion, ROS production, degranulation, and development of K/B×N arthritis (16). These results suggested that PTPN22 promotes, rather than inhibits, neutrophil-driven inflammation. On the other hand, the *in vitro* data from overexpression studies in cell lines suggested that PSTPIP2-bound PEST-PTPs suppress pro-inflammatory signaling (14). While the data from *in vivo* models are generally more reliable than cell line overexpression studies, in this case the single deficiency in the mouse model could have revealed only the unique role of PTPN22, while the functions, where it is redundant with other PEST-PTPs, could have remained hidden, leaving the overall role of PEST-PTPs in neutrophil-mediated inflammatory response unclear.

Another known binding partner of PSTPIP2, SHIP1 (14) is a multifunctional protein expressed predominantly by hematopoietic cells and osteoblasts. SHIP1 removes the 5’ phosphate from the product of PI3-kinase, PtdIns(3,4,5)P3, to generate PtdIns(3,4)P2 and this way partially antagonizes PI3-kinase pathway (17). SHIP1 deficiency results in enhanced ROS production and in reduced migration of neutrophils as a consequence of increased cell adhesion (18). *In vivo*, SHIP1-deficiency in mice causes inflammatory disease with myeloid infiltrates to the lungs and other organs (19). These data are generally consistent with anti-inflammatory function of SHIP1 and with connection to PSTPIP2. However, despite some common features, there are also many differences between the consequences of SHIP1 and PSTPIP2 deficiency and it has been unclear which functions of SHIP1 depend on their interaction.

To understand the *in vivo* function of PSTPIP2 interactions with PEST-PTPs or SHIP1, we have investigated the effects of mutations in PSTPIP2 that prevent binding of PEST-PTPs or SHIP1 *in vivo*. We have established the links between PSTPIP2 interactions, dysregulations of the pro-inflammatory pathways and disease symptoms and identified the functions of PEST-PTPs and SHIP1 in the context of their interactions with PSTPIP2 and autoinflammation.

## 2 Materials and methods

### 2.1 Mice

**Figure 1:**
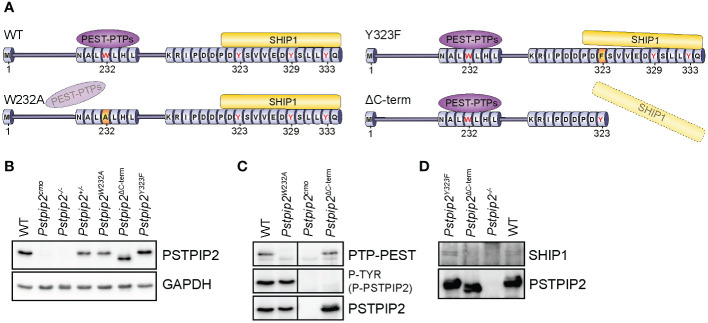
PSTPIP2 mutations in mice and verification of their effects on the interactions with binding partners. **(A)** Schematic representation of mutant PSTPIP2 proteins and their interactions in the individual mouse strains. **(B)** Lysates of purified neutrophils from WT and PSTPIP2 mutant mice were subjected to immunoblotting with the indicated antibodies to detect PSTPIP2 protein levels in the individual mouse strains. **(C)** PSTPIP2 was immunoprecipitated from the lysates of bone marrow cells from the indicated mouse strains. Co-immunoprecipitated PTP-PEST and PSTPIP2 tyrosine phosphorylation were detected by immunoblotting with PTP-PEST and phosphotyrosine antibodies, respectively. **(D)** Similar experiment as in **(C)** to detect interaction of PSTPIP2 with SHIP1. To maximize PSTPIP2 phosphorylation, cells in **(D)** were treated with pervanadate.

*Pstpip2^cmo^* mice on C57Bl/6NCrl genetic background carrying the c.293T→C mutation in the *Pstpip2* gene, were generated from C.Cg-*Pstpip2^cmo^*/J mouse strain on Balb/C genetic background (4, 5) obtained from The Jackson Laboratory (Bar Harbor, ME), by backcrossing for more than 10 generations to C57Bl/6J (7) and then for more than 5 generations to C57Bl/6NCrl. C57BL/6NCrl and C57BL/6J inbred strains were obtained from the animal facility of Institute of Molecular Genetics, Czech Academy of Sciences (Prague, Czech Republic). To generate mouse strains carrying mutations in the C-terminal part of the *Pstpip2* gene (*Pstpip2^Y323F^*, *Pstpip2^ΔC-term^*, and Pstpip2^Y323*^), specific guide RNA recognizing exon 14 of *Pstpip2* gene (5′-AGATGATCCTGATTACTCTG-3′) was designed and off-target analysis was performed using the online software CRISPOR Design Tool (http://crispor.tefor.net/). Cas9 protein and gRNAs with corresponding ssDNA template (5′-CCAGGCAGGTTAATGACTCTTACCACCTCTGACGTCACTGgaAGAGCAAACTGaAATCTTCAACCACActaaaATCcGGATCATCTGCAAAGGGAAGGGCACAGGACAGAACTCAGC-3′) were used for a zygote electroporation as described elsewhere (20). Similarly, mouse strains *Pstpip2^W232A^* and *Pstpip2^-/-^* were prepared by electroporation of gRNA recognizing exon 10 of *Pstpip2* (5′-ACTTCTTCCGGAATGCACTG-3′) together with a corresponding ssDNA template (5´-CATTTGCGACACATTGTTGTGACAGCTGATTCAGATGCAAtgcCAaTGCATTCCGGAAGAAGTTGATTCGTTCACATTCCTGAGCC-3´) (**Figure 1A**). Each strain was then backcrossed to C57BL/6NCrl background for more than 5 generations. Unless indicated otherwise, age of animals ranged from 8 to 12 weeks. Experiments in this work conducted on animals were approved by the Expert Committee on the Welfare of Experimental Animals of the Institute of Molecular Genetics and by the Czech Academy of Sciences and were in accordance with local legal requirements and ethical guidelines.

### 2.2 Micro computed tomography

**Figure 2:**
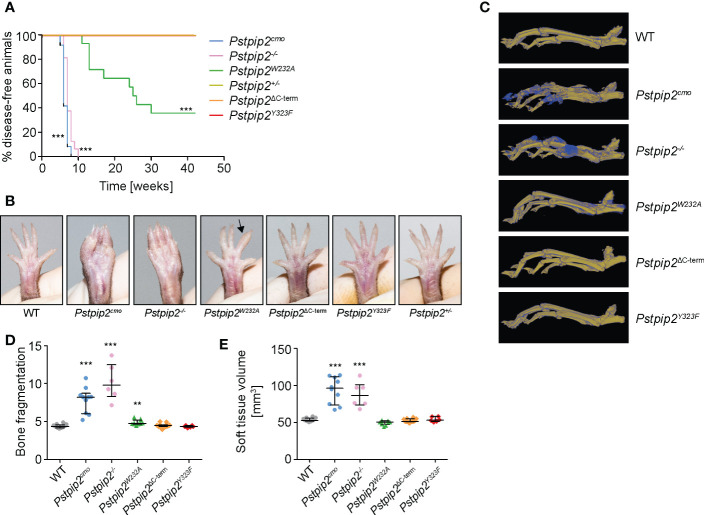
Symptoms of osteomyelitis in mutant mouse strains. **(A)** Mice of the indicated mouse strains were monitored for the onset of disease symptoms. The graph shows percentages of animals that were free of visible symptoms at the given time-point. Animal numbers and sexes in this experiment were as follows: *Pstpip2^+/-^* [6 males (m), 6 females (f)], *Pstpip2^-/-^* [7 m, 9 f], *Pstpip2^cmo^* [3 m, 11 f], *Pstpip2^W232A^* [4 m, 10 f], *Pstpip2^ΔC-term^* [5 m, 9 f], *Pstpip2^Y323F^* [2 m, 12 f]. **(B)** Photographs of hind paws of WT and mutant mice. 18-27 weeks old WT, *Pstpip2^cmo^*, *Pstpip2^-/-^*, and *Pstpip2^W232A^*, are compared to more than 50 weeks old *Pstpip2^ΔC-term^*, *Pstpip2^Y323F^*, and *Pstpip2^+/-^* mice. **(C)** Micro-CT reconstructions of hind paw bones of WT and mutant mice. Pseudocolors mark old (in yellow) and newly formed (in blue) bone mass. **(D)** Quantification of bone damage measured as bone fragmentation in paw bones of multiple mice detected in micro-CT scans. **(E)** Calculation of soft tissue volume from micro-CT scans as a measure of soft tissue swelling. Error bars represent median with interquartile range. Asterisks describe p values for comparisons with *Pstpip2^+/-^* **(A)** or WT **(D, E)**; **p ≤ 0.01, ***p ≤ 0.001. See *Materials and Methods* for further details on statistical analysis.

Hind paws of 3-5 mice per strain (16-25 weeks old) were used for the micro-CT analysis. They were scanned *in vivo* in X-ray micro-CT Skyscan 1176 (Bruker, Belgium) using the following parameters: voltage: 50 kV, current: 250 µA, filter: 0.5 mm aluminium, voxel size: 8.67 µm, exposure time: 2 s, rotation step: 0.3° for 180° total, object to source distance: 119.271 mm, and camera to source distance: 171.987 mm, time of scanning: 26 min. Virtual sections were reconstructed in NRecon software 1.7.1.0 (Bruker, Belgium) with following parameters: smoothing = 3, ring artifact correction = 4, and beam hardening correction = 36%. Intensities of interest for reconstruction were in the range from 0.0045 to 0.0900 Attenuation units. Same orientation of virtual sections was achieved with the use of the DataViewer 1.5.4 software (Bruker, Belgium). Micro-CT data analysis was performed using CT Analyser 1.18.4.0 (Bruker, Beelgium). Scans affected by technical artifacts resulting from spontaneous movements of animals were excluded from the analysis. Only distal half of the paws (from the half of the length of the longest metatarsal bone to fingertips) were analyzed. Bone damage (**Figure 2D**) is represented by bone fragmentation, which is calculated as the average number of bony objects (i.e. the bone with high density) per one virtual section. Without bone damage, 4-5 bone fragments (i.e. objects) per section are typically observed. With bone damage, this number increases. To calculate the volume of the soft tissue (**Figure 2E**), volumes of both high density and low density (newly formed) bone were subtracted from the volume of the entire paw (without background and noise).

### 2.3 Superoxide detection

Superoxide production *in vitro* was assessed by luminol-based chemiluminescence assay (21, 22). BM cells in IMDM supplemented with 0.2% FCS were plated at a density of 10^6^ cells per well into a black 96-well plate in duplicates (SPL Life Sciences, Naechon-Myeon, Korea). Cells were rested for 10 min at 37˚C and 5% CO_2_. Then, luminol (123072, Sigma-Aldrich) at final concentration 100 mM and silica (S5631, Sigma-Aldrich) 50 mg/cm^2^ were added. Luminescence was measured immediately on an EnVision plate reader (Perkin Elmer, Waltham, MA); each well was scanned every minute for 60 min.

### 2.4 Real-time quantitative PCR

RNA from neutrophils purified by negative selection was isolated with Zymo Research Quick-RNA Miniprep Plus Kit. The reverse transcription was performed with RevertAid First Strand cDNA Synthesis Kit (ThermoFisher Scientific). Real-time quantitative PCR was carried out using LightCycler 480 SYBR Green I Master mix (Roche) on Roche LightCycler 480 II instrument. The following primers were used (5’-3’):

*Cxcl2* AGTTTGCCTTGACCCTGAAGCC, CCAGGTCAGTTAGCCTTGCCTTTG;

*Actb* (β-actin) GATCTGGCACCACACCTTCT, GGGGTGTTGAAGGTCTCAAA;

*Pstpip2* CGGACTTGCTCATACATCTC, CTGGCAGAGTGAACACATTA.

### 2.5 Antibodies

Rabbit monoclonal antibodies to murine IL-1β (clone D3H1Z), neutrophil elastase (clone E8U3X), PTP-PEST (clone D4W7W), and rabbit polyclonal antibody to SHIP1 (D1163) were from Cell Signaling Technology, rabbit polyclonal antibody to GAPDH (#G9545) from Sigma-Aldrich. The monoclonal antibodies to phosphotyrosine (clone 4G10) and PSTPIP2 (clones PSTPIP2-01 and PSTPIP2-03 (14)) were produced in-house with the use of respective hybridomas. Flow cytometry antibodies Ly6G-FITC (catalog # 127606, also used for Western blot), Ly6C-PE-Cy7 (# 128018), CD11b-PE (# 101208) were from Biolegend and CD62L-APC (# 177-0621-81) was from eBioscience (ThermoFisher).

### 2.6 Cell isolation and activation

Hind paw leukocytes were isolated by crushing the tissue using mortar and pestle in PBS with 2% FCS. The resulting suspension was filtered over the cell strainer, followed by centrifugation (500 x g, 5 min, 2°C) and erythrocyte lysis in ACK buffer (150 mM NH_4_Cl, 0.1 mM EDTA (disodium salt), 1 mM KHCO_3_). Bone marrow cells were isolated by flushing femurs (cut at extremities) with PBS supplemented with 2% FCS, followed by red blood cell lysis with ACK buffer. Neutrophils were isolated from bone marrow cells by negative selection using mouse Neutrophil Isolation Kit (Miltenyi Biotec, catalog # 130-097-658) and autoMACS Pro magnetic cell separator (Miltenyi Biotec) according to manufacturer’s instructions. For LPS activation, 2×10^6^ cells in 700 µL IMDM with 0.1% FCS were placed in low protein-binding microcentrifuge tubes (Thermo Fisher Scientific). Subsequently, the cells were activated with 10 ng/ml LPS (L4516, Sigma-Aldrich) for 3 hours at 37˚C, 5% CO_2_. For pervanadate activation, pervanadate was prepared by mixing 10 mM sodium orthovanadate with 0.3% hydrogen peroxide followed by 20 min incubation at room temperature. 100 µl of the resulting mixture was used for activation of 1.2 × 10^7^ cells in 1 ml media (20 min at 37°C).

### 2.7 Cell lysis, and immunoprecipitation

For immunoblotting cell suspensions described above were lysed by addition of an equal volume of a 2× concentrated SDS-PAGE sample buffer (128 mM Tris [pH 6.8], 10% glycerol, 4% SDS, 2% DTT), followed by the sonication and heating (99˚C for 2 min). For immunoprecipitation cells were lysed in lysis buffer (50 mM TRIS-HCl pH 7.5; 150 mM NaCl; 1% *n*-dodecyl β-d-maltoside; 1000× diluted Diisopropyl-fluorophosphate [Sigma, Merck]; cOmplete EDTA-free protease inhibitor cocktail (Roche), PhosStop phosphatase inhibitor cocktail (Roche) at 1.2 × 10^8^ cells in 1.2 ml, for 30 min on ice. Post-nuclear supernatants were then incubated for 1 h with PSTPIP2-03 antibody (4.5 μg), followed by 1.5 h of incubation with 40 µl Protein A/G Plus agarose bead suspension (Santa Cruz Biotechnology) at 4°C. After washing on spin columns (Micro Bio-Spin columns, Bio-Rad Laboratories), immunoprecipitates were eluted with 30 μl SDS-PAGE sample buffer.

### 2.8 Tissue homogenates

Hind paw tissue was cut into small pieces and homogenized with Avans AHM1 Homogenizer (30 s, speed 25) in 1 ml RIPA buffer (TRIS-HCl pH7.5, 150 mM NaCl, 1% NP-40, 1% Deoxycholate, and 0.1% SDS, 5 mM iodoacetamide, 100× diluted Protease Inhibitor Cocktail set III [Calbiochem]). After two rounds of centrifugation (each 20,000 x g, 5 min, 2°C) the lysates were snap-frozen in liquid nitrogen and stored in -80°C.

### 2.9 ELISA

Frozen tissue homogenates (from 12-25 weeks old mice) described above were thawed, total protein concentration was measured using Pierce BCA Protein Assay Kit (Thermo Scientific #23227) and the samples were adjusted to equal protein concentration. ELISA was performed according to manufacturer’s instructions using IL-1 beta Mouse Uncoated ELISA Kit, MIP-2/CXCL2 Mouse ELISA Kit, MIP-1a (CCL3) Mouse Uncoated ELISA Kit (Invitrogen, ThermoFisher Scientific, catalog numbers 88-7013-88, EMCXCL2, and 88-56013-88), Mouse IL-17A/F Heterodimer DuoSet ELISA, and Mouse CXCL1/KC DuoSet ELISA DY5390-05, and DuoSet ELISA Ancillary Reagent Kit 2 (R&D Systems, catalog numbers DY5390-05, DY453-05, DY008).

### 2.10 Flow cytometry

Single-cell suspensions were labeled with 100× - 200× diluted antibodies and Hoechst 33342 dye (to detect dead cells) in PBS/2%FCS for 40 min on ice. Cells were then washed in PBS/2% FCS and analyzed on a BD Symphony flow cytometer. The data were analyzed with FlowJo software (BD Biosciences, Franklin Lakes, NJ).

### 2.11 Statistical analysis

**Figure 3:**
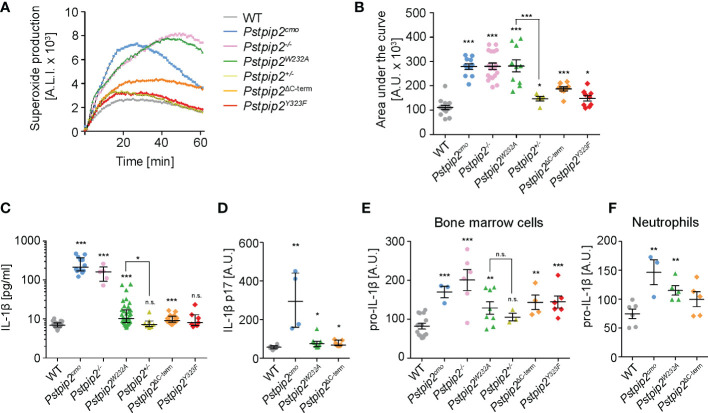
Production of superoxide and pro-IL-1β by neutrophils from WT and mutant mouse strains. **(A)** Superoxide production by silica-stimulated bone marrow cells measured in 1-min intervals by luminol-based chemiluminescence assay. **(B)** Quantification of the area under the curve for superoxide production measurements performed as in **(A)** on bone marrow cells from multiple mice. **(C)** IL-1β concentration in hind paw lysates detected by ELISA. **(D)** Quantification of active IL-1β p17 in hind paw lysates detected by Western blot. **(E, F)** Pro-IL-1β production by bone marrow cells **(E)** or purified neutrophils **(F)** activated with 10 ng/ml LPS detected by immunoblotting. Error bars represent mean ± SEM in **(B, E, F)** and median with interquartile range in **(C, D)**. Asterisks above individual columns describe p values for comparisons with WT, asterisks above connecting lines describe p values for comparisons of the columns connected by these lines; *p ≤ 0.05, **p ≤ 0.01, ***p ≤ 0.001, n.s. not significant. See *Materials and Methods* for further details on statistical analysis.

**Figure 4:**
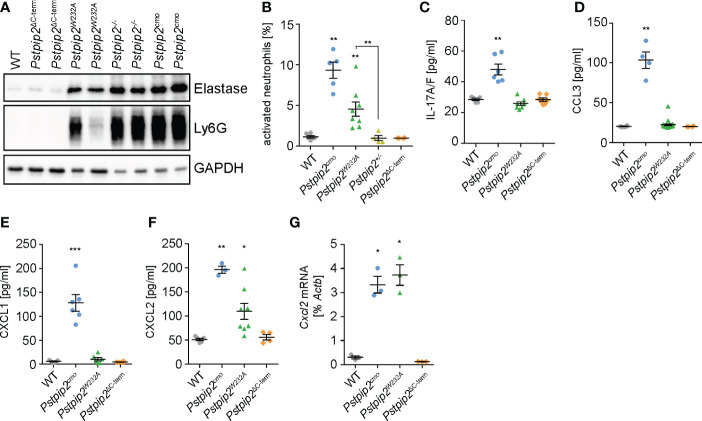
Neutrophil recruitment to the sites of inflammation. **(A)** Detection of neutrophil elastase and Ly6G in the hind paw lysates as a hallmark of neutrophil presence. **(B)** Percentages of activated (CD62L^-^) neutrophils within total neutrophils isolated from hind paws of the mice of indicated mouse strains measured by flow cytometry. **(C-F)** Concentrations of IL-17A/F **(C)**, CCL3 **(D)**, CXCL1 **(E)**, and CXCL2 **(F)** in hind paw lysates detected by ELISA. **(G)** *Cxcl2* mRNA levels in neutrophils purified from hind paws of the mice of indicated mouse strains determined by quantitative RT-PCR. Error bars represent mean ± SEM. Asterisks above individual columns describe p values for comparisons with WT, asterisks above connecting lines describe p values for comparisons of the columns connected by these lines; *p ≤ 0.05, **p ≤ 0.01, ***p ≤ 0.001. See *Materials and Methods* for further details on statistical analysis.

The p values were calculated in GraphPad Prism software (GraphPad Software, La Jolla, CA) using one-way ANOVA with *post-hoc* t-test for data in **Figures 3B, E, F**, **4B-G** (with Welch’s correction where variances were unequal) or Kruskal-Wallis test with *post-hoc* Mann-Whitney test for data with non-normal distribution (**Figures 2D, E**, **3C-D**). For multiple comparisons, significance threshold was adjusted with Holm-Bonferroni method. The p values for disease-free curves (**Figure 2A**) were calculated using the long-rank (Mantel-Cox) test.

## 3 Results

### 3.1 Generation of mouse strains with mutations in *Pstpip2*

To investigate the role of the interactions between PSTPIP2 and PEST-PTPs or SHIP1, we employed CRISPR/Cas9 technology to generate mutant mouse strains harboring mutations in PSTPIP2 that abrogate these interactions. Binding to PEST-family phosphatases is known to require W232, while binding to SHIP1 is dependent on C-terminal tyrosines (Y323, Y329, Y333) (**Figure 1A**). First, we generated mouse strain where W232 was replaced with alanine to prevent interaction with PEST phosphatases (*Pstpip2^W232A^*)(**Figure 1A** and **Supplementary Figure 1A**). In addition we attempted to generate a strain where all three C-terminal tyrosines were replaced with phenylalanines. Our targeting strategy was expected to also result in various truncations in the PSTPIP2 C-terminus. While the attempt to generate triple tyrosine mutant was unsuccessful, we obtained a strain, where a single nucleotide insertion into the codon of the first C-terminal tyrosine (Y323) created a stop codon resulting in the loss of the last twelve amino-acids (323–334), including all three targeted tyrosines (Pstpip2^Y323*^) (**Supplementary Figure 1B**). In addition we also obtained a strain where a 17 bp deletion resulted in a stop codon immediately after the first of the three tyrosines, Y323 (*Pstpip2^ΔC-term^*) (**Figure 1A** and **Supplementary Figure 1C**). In this strain, while Y323 is preserved, the absence of the amino acids immediately following it was expected to result in a loss of SHIP1 SH2 domain binding, because the amino acids downstream of the phosphorylated tyrosine are critical for this interaction (23–25). Thus, we expected that in this strain PSTPIP2 C-terminus including all the C-terminal tyrosines was effectively non-functional. Finally, within this attempt we obtained an additional strain where Y323 is replaced with phenylalanine (*Pstpip2^Y323F^*) (**Figure 1A** and **Supplementary Figure 1C**). In addition we generated mouse strain with 116 bp deletion encompassing part of the exon coding W232 together with a part of the preceding intron, resulting in the complete loss of PSTPIP2 expression (*Pstpip2^-/-^*) (**Supplementary Figure 1D**). Alignments of mutant and wild-type sequences are shown in **Supplementary Figures 1A–D**.

While PSTPIP2 protein levels were normal in *Pstpip2^Y323F^* mouse strain (**Figure 1B**), other mutations we introduced influenced its protein expression levels in neutrophil granulocytes. This was most evident in Pstpip2^Y323*^ mice where we detected only very low amounts of PSTPIP2 protein, ca 20 - 25% of wild-type levels (**Supplementary Figure 1E**). Interestingly, in *Pstpip2^ΔC-term^* mice, where the stop codon was only one position downstream, PSTPIP2 expression was comparable to wild-type (WT) mice. Since it was not possible to distinguish the effects of reduced PSTPIP2 expression on disease development from the effects of the mutation, we excluded Pstpip2^Y323*^ from subsequent analysis. Expression levels of *W232A* mutant were also somewhat reduced. However, they were comparable to PSTPIP2 expression in *Pstpip2^+/-^* heterozygotes (**Figure 1B**). Hence, we included *Pstpip2^+/-^* mice in subsequent analysis to control for the effects of reduced PSTPIP2 expression on the phenotype of *Pstpip2^W232A^* mice. PSTPIP2 protein was not detected in *Pstpip2^cmo^* and *Pstpip2^-/-^* neutrophils (**Figure 1B**). Interestingly, *Pstpip2* mRNA levels were normal in *Pstpip2^cmo^* neutrophils, while only traces of *Pstpip2* mRNA could be detected in *Pstpip2^-/-^* cells (**Supplementary Figure 1F**).

### 3.2 *Pstpip2* mutations abolish binding to PTP-PEST and SHIP1

To verify that the mutations had the intended effect and abolished interactions with major PSTPIP2 binding partners, we immunoprecipitated PSTPIP2 from bone marrow cells isolated from the individual mouse strains, followed by detection of PTP-PEST, SHIP1 and (phospho-)PSTPIP2 by immunoblotting. W232A mutation resulted in the loss of PTP-PEST binding without affecting PSTPIP2 phosphorylation, suggesting that PEST-PTPs bound to PSTPIP2 do not control its phosphorylation (**Figure 1C**). Conversely, the deletion of PSTPIP2 C-terminus resulted in the loss of PSTPIP2 phosphorylation, but it did not affect binding to PTP-PEST (**Figure 1C**). As expected, deletion of PSTPIP2 C-terminus also resulted in the loss of SHIP1 binding. Interestingly, mutation of a single C-terminal tyrosine Y323 did not have any effect on SHIP1 binding (**Figure 1D**).

### 3.3 Mutation of W232 results in symptomatic disease, while mutations of the PSTPIP2 C-terminus do not cause disease symptoms

Each mutant mouse strain was monitored for the development of chronic multifocal osteomyelitis symptoms (**Figure 2A**). *Pstpip2^-/-^* and *Pstpip2^cmo^* mice developed the first visually observable symptoms within 8 weeks after birth. In *Pstpip2^W232A^* strain the first symptom occurrence was delayed till 14-16 weeks of age. Only hind paws were affected in this strain. Visible kinks or swelling in the tails were not detected. In addition, the disease was much milder and not 100% penetrant since part of the animals remained asymptomatic throughout the entire 42 weeks of observation. The mutations of the PSTPIP2 C-terminus did not result in any visually detectable symptoms. The same was true for heterozygous *Pstpip2^+/-^* mice (**Figures 2A, B**).

In agreement with these results, micro-CT data (**Figure 2C**) showed significantly elevated bone damage in *Pstpip2^cmo^*, *Pstpip2^-/-^*, and *Pstpip2^W232A^* mice, while in the animals carrying mutations in the C-terminus (*Pstpip2^ΔC-term^*, *Pstpip2^Y323F^*), no bone damage was detected (**Figure 2D**). Compared to *Pstpip2^cmo^* and *Pstpip2^-/-^*, the bone damage in *Pstpip2^W232A^* animals was very mild, though still significantly increased. Soft tissue volume, a measure of swelling, was significantly higher only in *Pstpip2^cmo^* and *Pstpip2^-/-^* mice (**Figure 2E**). No significant increase of soft tissue swelling was detected in *Pstpip2^W232A^* animals. Taken together, the interaction with PEST-PTPs abrogated by W232A mutation plays more important role in the control of inflammation than binding of SHIP1, the loss of which does not contribute to the development of visible symptoms.

### 3.4 Differential control of ROS and IL-1β production by PSTPIP2 binding partners

Two key pro-inflammatory pathways are known to be dysregulated in *Pstpip2^cmo^* neutrophils, pathway leading to the activation of NADPH oxidase and pathway stimulating production of IL-1β. While IL-1β overproduction triggers spontaneous inflammation, superoxide production by NADPH oxidase is critical for the bone damage (8). Therefore, we aimed to evaluate whether the interactions of PSTPIP2 with PEST-PTPs and SHIP1 control these pathways and, consequently a pathogenesis of CMO. Consistent with previously published data, we observed substantially increased ROS production by *Pstpip2^cmo^* and *Pstpip2^-/-^* cells upon silica stimulation. Strikingly, in *Pstpip2^W232A^* cells ROS production was deregulated to a similar extent as in *Pstpip2^cmo^* and *Pstpip2^-/-^* cells. On the other hand, cells from mice that do not develop any visible disease symptoms, including *Pstpip2^ΔC-term^*, *Pstpip2^Y323F^* and *Pstpip2^+/-^* showed only minor elevation of ROS production (**Figures 3A, B**). To test the activity of IL-1β pathway, we measured the concentration of IL-1β in the lysates from hind paws of WT and mutant mice. IL-1β levels measured by ELISA were significantly increased in *Pstpip2^cmo^*, *Pstpip2^-/-^*, *Pstpip2^W232A^*, and *Pstpip2^ΔC-term^* mice. However, in contrast to the ROS production, IL-1β pathway dysregulation was milder in *Pstpip2^W232A^* and *Pstpip2^ΔC-term^* mice, when compared to the strains completely lacking PSTPIP2 protein (**Figure 3C**). We have also performed an analysis of active form IL-1β p17 in the strains that had abrogated binding between PSTPIP2 and its interacting partners. It showed similar results with the highest increase in *Pstpip2^cmo^* mice and a small increase in *Pstpip2^W232A^* and *Pstpip2^ΔC-term^* mice (**Figure 3D**). Next, we sought to evaluate the *in vitro* ability of isolated bone marrow cells from these mutants to produce pro-IL-1β upon LPS stimulation. Consistent with the *in vivo* results, the highest pro-IL-1β production was observed in *Pstpip2^cmo^* and *Pstpip2^-/-^* cells while *Pstpip2^W232A^* and *Pstpip2^ΔC-term^* displayed only moderate increase (**Figure 3E**). Similar results were also obtained with purified bone marrow neutrophils, although in *Pstpip2^ΔC-term^* mice the increase in pro-IL-1β production was not statistically significant (**Figure 3F**). These observations suggest that PSTPIP2-bound PEST-PTPs play dominant role in the control of the oxidative burst, while the regulation of IL-1β production is more equally divided between the both PSTPIP2 binding partners.

### 3.5 PEST-PTPs regulate neutrophil recruitment to the site of inflammation

Neutrophils, a critical cell type in osteomyelitis development in *Pstpip2^cmo^* mice, were found to infiltrate the sites of inflammation in these animals. To assess the extent of neutrophil infiltration, we detected neutrophil markers neutrophil elastase and Ly6G in the lysates prepared from hind paws of WT and mutant animals. Increased presence of these markers was detected in *Pstpip2^cmo^*, *Pstpip2^-/-^*, and *Pstpip2^W232A^* tissues even in the absence of visible symptoms, but not in *Pstpip2^ΔC-term^* (**Figure 4A**). These data confirm the involvement of neutrophils in the development of sterile inflammation. They also document the importance of PSTPIP2 binding to PEST-PTPs, which prevents neutrophil accumulation in the affected tissues. To further assess the activation status of these neutrophils, we measured the levels of CD62L on neutrophils isolated from hind paws of these animals. CD62L is shed as a result of neutrophil activation and lost from neutrophil surface (26). In *Pstpip2^cmo^* mice, the proportion of activated (CD62L^-^) neutrophils was significantly increased, when compared to the WT animals. Strikingly, group that contained both symptomatic and asymptomatic *Pstpip2^W232A^* mice also showed significantly increased levels of neutrophil activation. No such increase was observed in *Pstpip2^ΔC-term^* mice (**Figure 4B**). Thus, PEST-PTP binding to PSTPIP2 is an important component of the mechanism controlling neutrophil activation and infiltration to the site of inflammation.

Chemokines play a key role in the neutrophil recruitment to the inflamed tissues (27). One of the factors able to control chemokine production in these tissues is IL-17 (28). Indeed, IL-17A/F levels were increased in the lysates prepared from footpads of *Pstpip2^cmo^* mice (**Figure 4C**). However, *Pstpip2^W232A^* and *Pstpip2^ΔC-term^* mice did not show any alterations. Thus, while in *Pstpip2^cmo^* mice IL17A/F could be contributing to the neutrophil recruitment to the inflammatory lesions, in *Pstpip2^W232A^* mice this recruitment appears to be IL17A/F independent. Next, we focused on the most prominent chemokines known to attract neutrophils to the sites of inflammation, including CCL3 (MIP-1α), CXCL1 (KC), and CXCL2 (MIP-2). Concentration of all three chemokines measured by ELISA in hind paw lysates was increased in *Pstpip2^cmo^* mice (**Figures 4D–F**). Importantly, only the concentration of CXCL2 was elevated in *Pstpip2^W232A^* mice and none in *Pstpip2^ΔC-term^* animals, suggesting that dysregulation of CXCL2 is responsible for the early neutrophil recruitment initiating the disease development. Production of the other chemokines, as well as IL-17A/F, may be triggered later on as a secondary effect of progressing inflammation. CXCL2 is known to be secreted by tissue resident cells, such as epithelial cells, fibroblasts, mast cells or macrophages (29–34). However, the data of Immunological Genome Project Consortium (35) showed a very high expression of CXCL2 in thioglycolate induced peritoneal neutrophils, raising the possibility that in the context of CMO, neutrophils could be a major source of this chemokine. Strikingly, neutrophils purified from footpads of *Pstpip2^cmo^* and *Pstpip2^W232A^* mice both showed similar substantially increased levels of *Cxcl2* mRNA. On the other hand, *Pstpip2^Δ-term^* neutrophils displayed levels comparable to their WT counterparts (**Figure 4G**). These results support the hypothesis that during CMO disease development, neutrophils are fueling their own recruitment via a positive feedback loop driven by production of CXCL2, which is further assisted by other chemokines in the later stages of the disease.

## 4 Discussion

During inflammatory response, neutrophils are capable of producing substantial collateral damage. It is exemplified by the development of autoinflammatory disease caused by the loss of PSTPIP2 adaptor protein, where hyper-activated neutrophils are the critical cell type required for inducing harm to the tissues (7, 8). Similar to other adaptor proteins, function of PSTPIP2 is mediated by its interactions with other signaling molecules. The most prominent include PEST-family PTPs and SHIP1 (12–14). However, their particular roles in the suppression of inflammation by PSTPIP2 have been unknown. Our data demonstrate the importance of the interactions of PEST-PTPs with PSTPIP2. The loss of their binding leads to the development of autoinflammatory disease, which is milder but otherwise similar to the disease that develops as a consequence of the inactivation of PSTPIP2 gene. The loss of interaction with SHIP1 does not result in visible disease symptoms. Nevertheless, certain level of immune system dysregulation can still be observed. These data suggest that both binding partners contribute to the suppression of pro-inflammatory signaling and autoinflammation. However, only the loss of PEST-PTP binding results in the dysregulation strong enough to cause visually observable disease symptoms.

Interestingly, PEST-PTPs play an important role in another autoinflammatory disorder named PAPA syndrome (pyogenic sterile arthritis, pyoderma gangrenosum, and acne) which is caused by the loss of their binding to PSTPIP1, a homologue of PSTPIP2. However the mechanism triggering the disease is very likely different. In PSTPIP1, the loss of PEST-PTP binding results in PSTPIP1 hyperphoshorylation and consequent hyperactivation of pyrin inflammasome (36). Here, we did not observe any changes in PSTPIP2 phosphorylation after the loss of PEST-PTP binding. Moreover, when we abrogated this phosphorylation by the deletion of PSTPIP2 C-terminus, we observed increase of pro-inflammatory markers, while if the analogy with PSTPIP1 were valid, we would rather expect the opposite.

Interestingly, the mouse strains, where interactions with PEST-PTPs or SHIP1 were abolished, both displayed similar level of dysregulation of IL-1β pathway. Thus, the observed differences in disease manifestation cannot be explained by differential IL-1β production. On the other hand, ROS production in *Pstpip2^W232A^* mice lacking PSTPIP2 - PEST-PTP interaction was deregulated to a similar extent as in mice entirely lacking PSTPIP2 protein, while the loss of SHIP1 binding in *Pstpip2^ΔC-term^* animals had only mild effect on ROS. These data suggested that deregulated ROS production determines whether the mice develop bone damage and visible disease symptoms. We have shown before that ROS production is critical for damage to the bones in mice lacking PSTPIP2 but it is not required for enhanced IL-1β production and soft tissue inflammation (8). In line with this finding, our microCT analysis did not detect any evidence of soft tissue swelling in *Pstpip2^W232A^* mice. Our data suggest that in mild variants of CMO disease, ROS production may decide between symptomatic and asymptomatic outcome and could be considered as a potential pharmacological target in treatment strategies for similar diseases in humans.

The milder dysregulation of IL-1β production in *Pstpip2^W232A^* than in *Pstpip2^-/-^* and *Pstpip2^cmo^* mice can potentially explain the milder phenotype observed in these mice. These data show that PEST-PTPs are not sufficient for PSTPIP2-mediated control of inflammation and other factors must play a role. Similar level of IL-1β dysregulation in *Pstpip2^ΔC-term^* mice suggests that SHIP1 can to some extent control IL-1β pathway and attenuate inflammatory response in *Pstpip2^W232A^* mice. Analysis of a mouse model with simultaneous loss of both binding sites in PSTPIP2 could help clarify the role of SHIP1, since IL-1β deregulation there may reach higher levels sufficient for the development of fully expressed symptoms. However, at present, such a model is not available. It is also possible that some other binding partners or features of PSTPIP2 are contributing to the control of inflammation in addition to PEST-PTPs and SHIP1.

The increased neutrophil infiltration in hind paws of *Pstpip2^W232A^* and PSTPIP2-fully deficient mouse strains but not in *Pstpip2^ΔC-term^* animals suggested that chemokine production regulating neutrophil recruitment could also be dysregulated and help explain differences in disease manifestation between *Pstpip2^W232A^* and *Pstpip2^ΔC-term^* animals. Indeed, we have observed increased amounts of CXCL1, CXCL2, and CCL3 in the hind paws of PSTPIP2 fully deficient mice. However only CXCL2 showed increased levels in *Pstpip2^W232A^* mice. Moreover, we detected high increase of *Cxcl2* mRNA expression in *Pstpip2^cmo^* and *Pstpip2^W232A^* neutrophils purified from the site of inflammation. This increase appeared higher (more than ten-fold) than overall increase in the inflamed tissues (less than four-fold). These data suggested that neutrophils are a major source of CXCL2 during CMO development. Increased activation of transcription factor NF-κB was demonstrated in *Pstpip2^cmo^* mice (37). Production of pro-IL-1β, which is elevated in these mice, as well as production of CXCL1, CXCL2 and CCL3 are driven by this transcription factor (37–45). This suggests that there could be a common pathway, which is dysregulated in *Pstpip2^cmo^* mice, leading to enhanced NF-κB activity followed by increased production of pro-IL-1β and the chemokines. In contrast to *Cxcl1* and *Ccl3*, *Cxcl2* gene expression is not negatively regulated by a transcription factor ATF3, which may explain increased sensitivity of *Cxcl2* gene expression to pro-inflammatory signaling and selective upregulation in *Pstpip2^W232A^* mice (46–48). CXCL2 production by neutrophils may represent an additional critical step in disease progression. It is very likely that activation of the positive feedback loops driven by neutrophil-produced IL-1β and CXCL2 result in substantial amplification of neutrophil response via further neutrophil recruitment and secondary production of additional IL-1β, CXCL2 and other chemokines, propelling the disease to its symptomatic stage. Our results also suggest that chemokine networks together with IL-17 may represent potential pharmacological targets/biomarkers in similar diseases in humans. However, proper analysis of their role would require further testing using mice double deficient in *Pstpip2* and receptors or other critical components of these pathways.

In summary, together with earlier published results, our data demonstrate dysregulation of three major pathways, including production of IL-β, reactive oxygen species, and neutrophil-attracting chemokines, which jointly contribute to the development of CMO disease in *Pstpip2^cmo^* mouse model. Recruitment of PEST-PTPs and SHIP1 by PSTPIP2 have differential regulatory effects on these pathways. PEST-PTPs have a dominant role in the control of reactive oxygen species and to some extent also in the control of chemokine production, while they appear similarly important as SHIP1 in the control of IL-1β pathway. Direct targets of these phosphatases that regulate these pathways still remain unknown. However, the new mouse models generated within this work will be instrumental for their future identification.

## Data availability statement

The original contributions presented in the study are included in the article **Supplementary Material**. Further inquiries can be directed to the corresponding author.

## Ethics statement

The animal study was reviewed and approved by Expert Committee on the Welfare of Experimental Animals of the Institute of Molecular Genetics and the Czech Academy of Sciences.

## Author contributions

TB and RS conceived, designed and supervised the study. NP, ID, JK, MF, FS, JPr, PK, JPo, and TS performed experiments. NP, JK, FS, JPr, PK and TB analyzed and interpreted the data. NP and TB wrote the first draft of the manuscript. All authors contributed to the article and approved the submitted version.
