# Supplementary material for: Molecular interactions of adaptor protein PSTPIP2 control neutrophil-mediated responses leading to autoinflammation
Source: Front Immunol. 2022 Dec 20;13:1035226. doi: 10.3389/fimmu.2022.1035226 (PMC9807597; doi:10.3389/fimmu.2022.1035226)
Supplement: Supplementary file 1 [file Image_1.pdf]

Supplementary Material

Molecular interactions of adaptor protein PSTPIP2 control neutrophil-mediated responses leading to autoinflammation

Nataliia Pavliuchenko, Iris Duric, Jarmila Kralova, Matej Fabisik, Frantisek Spoutil, Jan Prochazka, Petr Kasparek, Jana Pokorna, Tereza Skopcova, Radislav Sedlacek, Tomas Brdicka*

*** Correspondence:** Tomas Brdicka: tomas.brdicka@img.cas.cz

Supplementary Figure

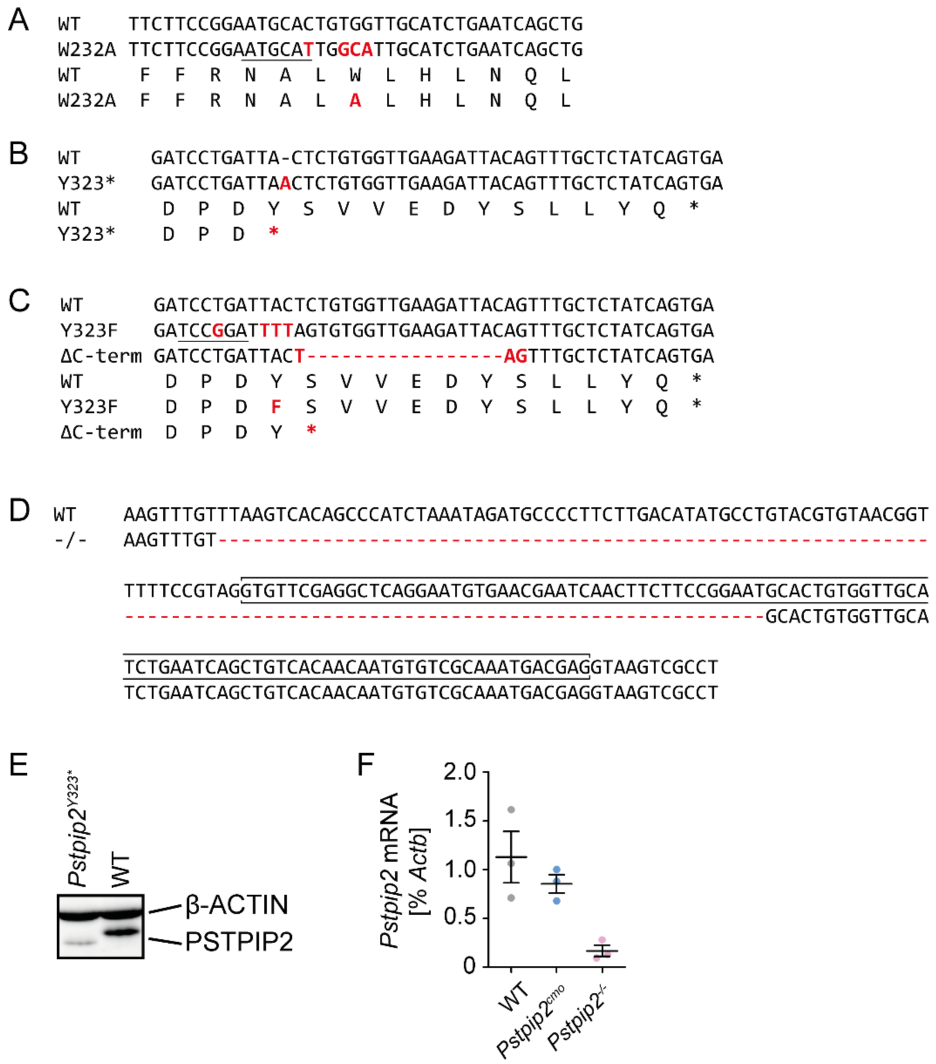

Supplementary Figure 1. (A-D). Alignments of WT and mutant nucleotide and amino acid sequences. The sequences were determined by Sanger sequencing of PCR products generated by amplification of the targeted regions from the genomic DNA. **(A)** *Pstpip2*^{W232A}; mutated nucleotides and corresponding amino acid are labelled in red, sequence of NsiI restriction cleavage site introduced for genotyping purposes is underlined. **(B)** *Pstpip2*^{Y323*}; inserted nucleotide is labelled in red, the resulting stop codon is at the amino acid level represented by red asterisk. **(C)** *Pstpip2*^{Y323F}, *Pstpip2*$^{\Delta C\text{-}term}$; mutated nucleotides and corresponding amino acid are labelled in red, sequence of BspEI restriction cleavage site introduced for genotyping purposes is underlined. Deletion is depicted as dashed red line, the resulting stop codon is labelled in red and at the amino acid level represented by red asterisk. **(D)** *Pstpip2*$^{-/-}$; deletion is depicted as dashed red line, affected exon is boxed. **(E)** PSTPIP2 protein expression in neutrophils from *Pstpip2*^{Y323*} mouse strain detected by immunoblotting. As a loading control β-ACTIN was stained on the same membrane. **(F)** *Pstpip2* mRNA level in WT, *Pstpip2*^{cmo}, and *Pstpip2*$^{-/-}$ neutrophils was determined by quantitative real-time PCR.
